# Retinal Electrical Synapses Prime Neighbor Cells to Amplify Looming Related Threat Signals and Support Escape Behavior

**DOI:** 10.21203/rs.3.rs-9903415/v1

**Published:** 2026-06-04

**Authors:** Béla Völgyi, Gergely Szarka, Arnau Sans-Dublanc, Bram Nuttin, Virág Tóth, Áron Kolozsvári, Tamás Kovács-Öller, Karl Farrow

**Affiliations:** University of Pécs; University of Pecs; Neuroelectronics Research Flanders; KU Leuven Center for Neuroscience; University of Pecs; University of Pecs; University of Pecs; VIB-KU Leuven Center for Neuroscience

**Keywords:** gap junction, electrical synapses, ganglion cell, inner plexiform layer, ganglion cell layer, retina, parallel signaling

## Abstract

Rapid detection of looming objects is essential for survival, yet single retinal ganglion cells have receptive fields that are smaller than many behaviorally relevant looming stimuli. This mismatch raises the question of how the retina represents an expanding threat as it moves beyond the receptive field of a single neuron. Here, we show that gap junctions extend looming-related activity across a coupled network of transient OFF-alpha retinal ganglion cells. Using multiscale electrophysiology, genetic and pharmacological perturbations, and behavioral assays, we found that looming stimuli recruit neighboring retinal ganglion cells through electrical synapses. Targeted recordings showed that activation of one transient OFF-alpha cell can prime neighboring cells, allowing them to respond when the expanding edge reaches their receptive fields. Disrupting gap junction coupling reduced retinal population recruitment, altered looming-evoked response patterns in the superior colliculus, and impaired escape behavior, while leaving visual acuity, depth perception intact, and lateral-motion responses intact. These findings show that electrical synapses transform local looming responses in the retina into a coordinated population signal that extends beyond single receptive fields and supports escape behavior.

## INTRODUCTION

Detecting looming objects is essential for survival, as it enables rapid defensive or escape responses^1–3^. Previous studies have shown that mouse transient OFF-alpha retinal ganglion cells (tOFFα RGCs) play a key role in detecting looming motion and transmitting this information to the superior colliculus (SC), which in turn drives defensive behaviors^1–8^. However, the receptive field of single tOFFα RGCs span only ~ 10° of visual space^4^, whereas looming stimuli that elicit defensive behaviors often expand across much larger regions of the visual world^1,7,8^. This spatial mismatch raises a central question of how the retina and early visual system represents an expanding threat as it moves beyond the receptive field of any one neuron.

In particular, for tOFFα RGCs it has been shown that laterally moving contrasts do not effectively activate spiking activity^4^. A tOFFα RGCs located away from the stimulus center does not experience the full expanding object, instead encountering an edge moving laterally across its receptive field. Thus, a looming object that grows beyond the receptive field of the initially activated cell should fail to recruit neighboring tOFFα RGCs unless an additional circuit mechanism changes their excitability. How does the retina recruit spatially offset cells while preserving the distinction between looming motion and lateral motion?

Electrical synapses provide a candidate mechanism for this recruitment as they can coordinate activity across neighboring retinal neurons. Gap junctions are common in retinal circuits and can shape population activity among ganglion cells and amacrine cells^9–13^. In the context of looming detection, such coupling could allow activity initiated near the center of an expanding object to prime neighboring cells before the edge reaches their receptive fields. This mechanism predicts that disrupting gap-junction signaling should reduce retinal population recruitment, alter the pattern of retinal neural input to the SC, and weaken escape behavior without causing a general loss of visual function.

Here, we show that looming stimuli recruit retinal ganglion cells through gap-junction-dependent priming that extends looming-related activity beyond the receptive field of a single neuron. Using multielectrode recordings in the retina and SC, targeted recordings from A tOFFα RGCs, and behavioral perturbations, we identify a coupled tOFFα-centered circuit in which electrical synapses prime neighboring cells during looming stimulation. Disrupting this coupling reduces recruitment of neighboring neurons, reshapes SC response patterns, and weakens escape behavior while sparing other visual functions.

## RESULTS

We tested whether gap-junction-mediated coupling expands looming-related signaling beyond single receptive fields and thereby supports downstream threat responses. We first asked whether looming stimuli recruit a distributed retinal ganglion-cell population through gap-junction coupling. We then examined whether tOFFα RGCs show electrical-synapse-dependent neighbor priming during looming stimulation. Finally, we asked whether disrupting retinal gap-junction coupling alters superior colliculus activity and looming-evoked escape behavior while sparing other visual functions.

### Looming Recruits a Distributed RGC Population through Cx36-dependent Coupling

**Figure 1:**
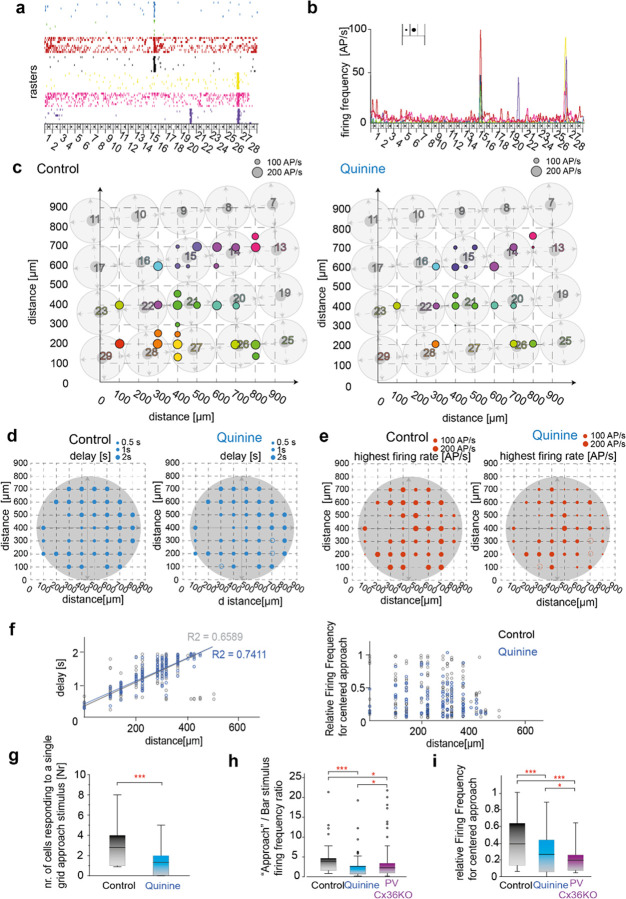
Gap junction dependent recruitment of RGCs during looming stimulation. **a)** Raster plot showing spiking activity of example RGCs recorded with a multielectrode array during repeated presentation of looming stimuli at different spatial locations. Stimuli were repeated 8 times per position. Numbers indicate stimulus positions within the stimulation matrix. **b)** Corresponding peristimulus time histograms (PSTHs) for the cells shown in (a). **c)**Spatial maps of RGC activity under control conditions (left) and during gap junction blockade with quinine (right). Electrode positions are indicated by grid intersections (100 μm spacing). Background pattern indicates stimulus locations. Circle size represents response magnitude, and colors correspond to stimulus identity. Multiple units recorded from the same electrode are shown as adjacent markers. **d, e)** Spatial distribution of RGC responses to a single expanding (looming) stimulus under control conditions and during gap junction blockade. **f)** Relationship between distance from the stimulus center and response latency (left) or normalized firing rate (right). **g)**Average number of RGCs activated per stimulus under control conditions and during gap junction blockade. **h)** Looming-to-bar response ratio (L/G ratio) under control conditions, gap junction blockade, and PV/Cx36KO conditions. **i)** Relative firing frequency of RGCs under control conditions, gap junction blockade (Ctrl = 0.42 ± 0.22, quinine = 0.27 ± 0.20), and PV/Cx36KO conditions (0.21 ± 0.16;). Shown are mean values (line), 25% and 75% quartiles (boxes), data range (whiskers).

We found that looming stimuli recruited a broad, distributed population of RGCs likely tuned to various visual attributes^14–16^. While looming detection has traditionally been attributed to specific circuits, our multi-electrode array (MEA) recordings in mouse retinas (n = 18) revealed that a expanding dark spot stimuli organized in a matrix fashion (Fig. 7; see methods) activated multiple units across the retinal surface, typically encompassing a heterogeneous mix of ON, OFF, and ON–OFF RGCs (Fig. 1a–d). We refer to these collectively as looming-sensitive cells, whereas looming-selective cells are those that in addition to sensing can also discriminate looming from lateral motion. To date only tOFFα RGCs meet this latter criterion^4^.

To reveal the mechanism behind this distributed recruitment we hypothesized that it depends on electrical synapse signaling which often coordinates population activity^10–12,17,18^. Indeed, pharmacological blockade of gap junctions using quinine (100 μM; N = 8 retinas, n = 1860 cells) markedly reduced looming responses in most cells (Fig. 1c) and decreased the average number of cells activated units per stimulus from 2.8 ± 1.9 under control conditions to 1.4 ± 1.2 (p < 0.001, Wilcoxon signed-rank test; Fig. 1g). Note, that a similar pharmacological blockade of GABAergic signaling increased the number of recruited RGCs and firing rates, indicating a fundamentally different role of inhibition in looming detection (**Supplementary Fig. 1**).

To confirm these observations under a simplified stimulus condition, we used a single expanding dark spot in MEA recordings. RGCs located near the center of the stimulus showed the shortest response latencies, whereas more distant cells responded with progressively longer delays (Fig. 1d, g). Contrary, RGC firing rates were less dependent on spatial position (Fig. 1e, f). The gap junction blockade (100 μM quinine) did not change response latencies but significantly reduced the total number of looming-sensitive cells (control: 388; quinine: 282) and their firing rates (p = 8.4 × 10^−^18, Wilcoxon signed-rank test; Fig. 1i). In addition, a similar reduction was observed in PV/Cx36KO mice (p < 0.001, Mann–Whitney U test), in which animals Cx36 gap junction contacts were genetically eliminated in parvalbumin expressing cells, including 8 RGC subtypes^4^.

To determine whether the recorded looming-sensitive population included looming-selective neurons, we calculated the ratio of responses to the looming stimulus and to drifting gratings (L/G ratio) for each cell. Under control conditions, the mean L/G ratio was 4.2 ± 1.5, with substantial variability across cells (L/G ranged between 1–22), consistent with the presence of multiple RGC subtypes. A pharmacological gap junction blockade reduced the mean L/G ratio to 2.5 ± 1.9 (p < 0.001, Wilcoxon signed-rank test). Moreover, a subset of RGCs (8.3 ± 0.7%) lost all looming-evoked spike responses while maintaining sensitivity to gratings, resulting in L/G < 1. Similar results in PV/Cx36KO mice (N = 5 retinas, n = 1463 cells; L/G ratio was significantly lower, 3.2 ± 2.9; p < 0.05, Mann–Whitney U test) than control counterparts confirming that gap junction-dependent recruitment is a robust feature of the looming response (Fig. 1h). This finding highlights a critical role for gap junctions in both the distribution of looming-mediated signals among a heterogeneous RGC population and the emergence of looming-sensitivity (Fig. 1h). Accordingly, the remainder of the study aims to provide a mechanistic understanding of the processes underlying this phenomenon.

### tOFFα RGCs Respond to Looming beyond the Receptive Fields

**Figure 2:**
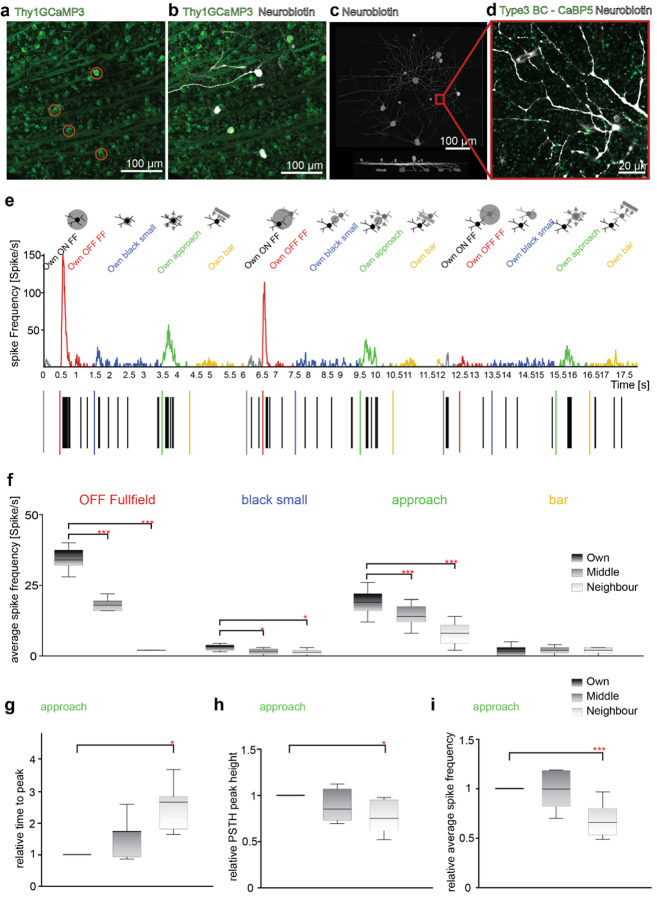
Neighbor stimulation evokes looming responses in tOFFα RGCs consistent with priming. **a)** Surface view of Thy1-GCaMP3 retina; red circles indicate targeted tOFFα RGCs. **b)**Thy1-GCaMP3 retina after Neurobiotin (NB) injection. **c)** Reconstruction of NB-filled tOFFα RGC array. **d)** Co-localization of tOFFα dendrites with type 3 bipolar cell axon terminals (CaBP5). Scale bars: a–c, 100 μm; d, 20 μm. **e)** Representative loose-patch recordings from a tOFFα RGC showing spike time stamps (bottom) and peristimulus time histograms (top) during four stimulus conditions: full-field dark spot, small dark spot, expanding spot (looming stimulus), and laterally moving bar. Stimuli were presented at three spatial locations: centered on the recorded cell, at the receptive-field periphery, and centered on a neighboring tOFFα RGC. Colored lines indicate stimulus onset. **f)** Spike-frequency responses of the recorded cell for each stimulus and spatial location. Responses decreased with stimulus displacement for full-field and small-spot stimuli, whereas looming responses remained detectable when the stimulus was centered on the neighboring cell. Moving-bar stimuli did not evoke reliable responses. **g–i)**Pooled data from multiple cells (n = 14). **g)** Response latency increased with stimulus displacement from the recorded cell (CC: 0.17 ± 0.08 s; NC: 0.38 ± 0.06 s; * p < 0.05; Wilcoxon signed-rank test). **h)** Relative peak response amplitude decreased significantly with increasing displacement, reflecting the reduced efficacy of peripheral stimulation (*** p<0.001, * p < 0.05; Wilcoxon signed-rank test). **i)**Relative spike frequency showed a similar reduction but remained detectable during neighbor stimulation. Shown are mean values (line), 25% and 75% quartiles (boxes), data range (whiskers). Statistical significance: *p < 0.05, ***p < 0.001, Wilcoxon signed-rank test.

We identified tOFFα RGCs that responded not only to looming stimuli centered on their own dendritic arbor, but also to those over neighboring tOFFα receptive fields. To investigate mechanisms underlying the gap junction mediated looming-selectivity observed in the MEA experiments, we performed patch-clamp and Ca^++^-imaging recordings in the Thy1-GCaMP3 mouse line and targeted tOFFα RGCs, the only known looming-sensitive cell in the retina. We targeted large, brightly labeled somata in the *in vitro* retina (Fig. 2a). Neurobiotin filling based soma/dendritic morphology, tracer coupling and dendritic co-stratification with CaBP5 (or HCN4) labeled type3a bipolar cell axon terminals confirmed tOFFα identity of targeted cells (Fig. 2b–d).

Recorded cells underwent a complex stimulation paradigm. The applied stimulus sequence included full-field, small-spot, looming, and moving-bar stimuli that were first centered over the receptive field of the recorded cell (central cell, CC) and then displaced toward a neighboring tOFFα RGC receptive field (neighbor cell, NC) (Fig. 2e; N = 4 retinas, n = 14 cells). Consistent with the known looming-selectivity of tOFFα RGCs^4^ the looming stimulus evoked robust spiking responses, whereas the laterally moving bar produced little or no response when stimuli were centered over the CC. Displacing the stimulus toward the periphery of the CC receptive field reduced responses to all stimuli, as expected from partial receptive-field activation. Interestingly, however, when the stimulation focus was further offset to cover the NC receptive field, the looming stimulus still evoked responses in the recorded CC, whereas full-field, small-spot, and moving-bar stimuli failed to do so (Fig. 2e, f). These results indicated that the activation of an adjacent cell can elicit responses in the recorded RGC, a process we termed neighbor priming. Moreover, this effect was stimulus-specific, as neighbor-centered responses were disproportionately stronger for looming compared to bar motion and stationary stimuli.

To further characterize the mechanism of neighbor priming, we performed a detailed comparison of CC responses evoked by looming stimuli only presented at three different spatial locations. Looming stimuli were first centered on the CC, then moved to the CC receptive-field periphery and finally focused on the NC receptive field (Fig. 2g–i; n = 14 cells). Under these conditions the NC-centered expanding spot could reach at most the CC receptive field periphery and resemble the inadequate laterally moving edge, an inadequate stimulus. As expected, the response latency progressively increased as the stimulus focus was displaced away from the CC receptive-field center. Compared to CC-centered stimuli, NC-centered looming stimulation elicited significantly longer latencies (Fig. 2g), indicating that the CC was not directly stimulated. On the other hand, the response magnitude (measured as PSTH peak amplitude and average spike frequency), although decreased significantly, remained detectable under all conditions (Fig. 2g, h). Together, these results indicate that CC responses during NC-centered stimulation cannot be explained solely by direct CC receptive-field activation but rather indicate the lateral signal flow between cells, further confirming the neighbor priming hypothesis.

### Gap Junction Blockade Abolishes Neighbor-evoked Looming Responses in tOFFα RGCs

**Figure 3:**
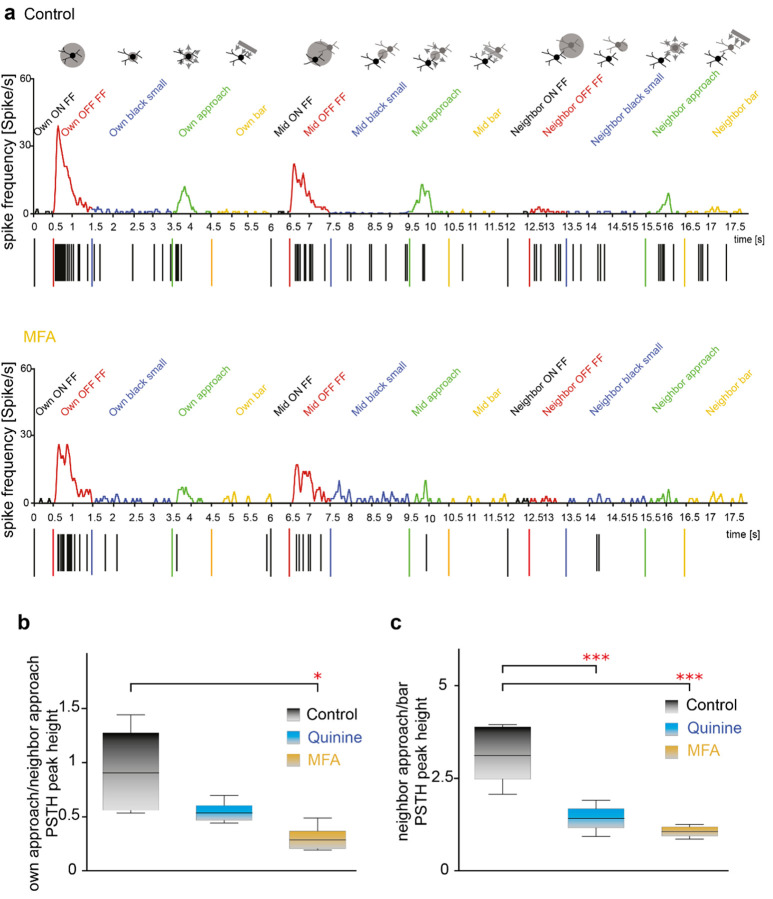
Neighbor-Evoked Light Responses of tOFFα Retinal Ganglion Cells are Gap Junction dependent **a)** Representative recordings from a tOFFα RGC showing responses to full-field, small-spot, looming, and moving-bar stimuli under control conditions (top) and after intracellular gap junction blockade with meclofenamic acid (MFA, 40 μM; bottom). Stimuli were presented at three spatial locations: centered on the recorded cell, at the receptive-field periphery, and centered on a neighboring RGC. Neighbor-evoked responses to the looming stimulus were strongly reduced following gap junction blockade. **b)**Quantification of response changes. Left: relative response amplitude (ratio of responses to direct versus neighboring receptive-field stimulation) decreased after MFA or quinine treatment (Ctrl: 0.9±0.52; Quinine: 0.55±0.09; MFA: 0.30±0.10). **c)** Looming-to-bar response ratio decreased following gap junction blockade with quinine or MFA (Ctrl: 3±0.8; Quinine: 1.4±0.44; MFA: 1.0±0.15;). Shown are mean values (line) in *b* and *c* 25% and 75% quartiles (boxes), data range (whiskers). Statistical significance: *p < 0.05, ***p < 0.001, Wilcoxon signed-rank test.

Pharmacological tests showed that a gap junction blockade markedly weakened the neighbor-evoked looming response in tOFFα RGCs. To show that the neighbor priming mechanism is gap junction mediated, we combined targeted tOFFα RGCs recordings (N = 4 retinas, n = 8 cells) with gap junction blockade pharmacology using meclofenamic acid (MFA; 40 μM) (Fig. 3a). The MFA was administered intracellularly *via* the recording pipette (see Methods for details and Szarka et al. 2024^19^) allowing for the blockade of gap junctions of the recorded cell while the surrounding network remained initially intact. Accordingly, the initial recordings (within ~ 5 minutes of seal formation) were handled as control measurements, while those obtained subsequently were considered under conditions of effective gap junction blockade.

Consistent with previous observations, CC stimulation of tOFFα RGCs evoked stronger responses when compared to those of NC stimulations across all stimulus types in control conditions. NC stimulation elicited activation of the recorded cell was most prominent when looming stimuli were presented (Fig. 3a). However, following a 2–4 minutes incubation with MFA, a substantial reduction in response amplitude was observed (Fig. 3a). This attenuation was already evident even during CC stimulation, but it was more pronounced for peripheral stimuli and nearly annihilated responses to NC stimulation (Fig. 3a). To confirm the involvement of gap junctions, we replicated the pharmacological blockade using quinine (N = 4 retinas, n = 6 cells), a connexin36 (Cx36)-selective gap junction antagonist^19–21^. Although quinine was generally less effective than MFA in suppressing RGC responses, it produced qualitatively similar reductions in tOFFα RGC activity.

To quantify the above effects, we computed a relative amplitude index, defined as the ratio of PSTH peak height for CC looming stimulation to that of NC looming stimulation, under both control and gap junction blocked conditions (MFA and quinine). We observed a significant (p **<** 0.05 Wilcoxon signed-rank test) reduction in the CCpeak/NCpeak ratio following gap junction blockade (Fig. 3b). This analysis quantitatively corroborates previous qualitative observations, demonstrating that both MFA and quinine elicited a pronounced reduction, or complete suppression, of the NC-driven looming response (Fig. 3b).

In a complementary analysis, we assessed the tOFFα RGC looming discrimination capacity at three spatial locations under both control conditions and following gap junction blockade with MFA (or quinine in some experiments). Consistently, we found that the disruption of gap junction signaling in tOFFα RGCs impaired looming detection and thereby reduced the ability to discriminate between the looming stimulus and the moving bar (L/G ratio) (Fig. 3c; p < 0.001 Wilcoxon signed-rank test). These results indicate that Cx36-dependent gap junction coupling is required for the full neighbor-evoked looming response in tOFFα RGCs.

Collectively, the in vitro functional data presented above indicate that gap junction–mediated neighbor priming in tOFFα RGCs extends looming coding beyond single receptive fields and plays a crucial role in the emergence of looming sensitivity. The remainder of this study will elucidate the consequences of this mechanism at both the network and behavioral levels.

#### Retinal Gap Junction Perturbation Reshapes SC Looming Responses

**Figure 4:**
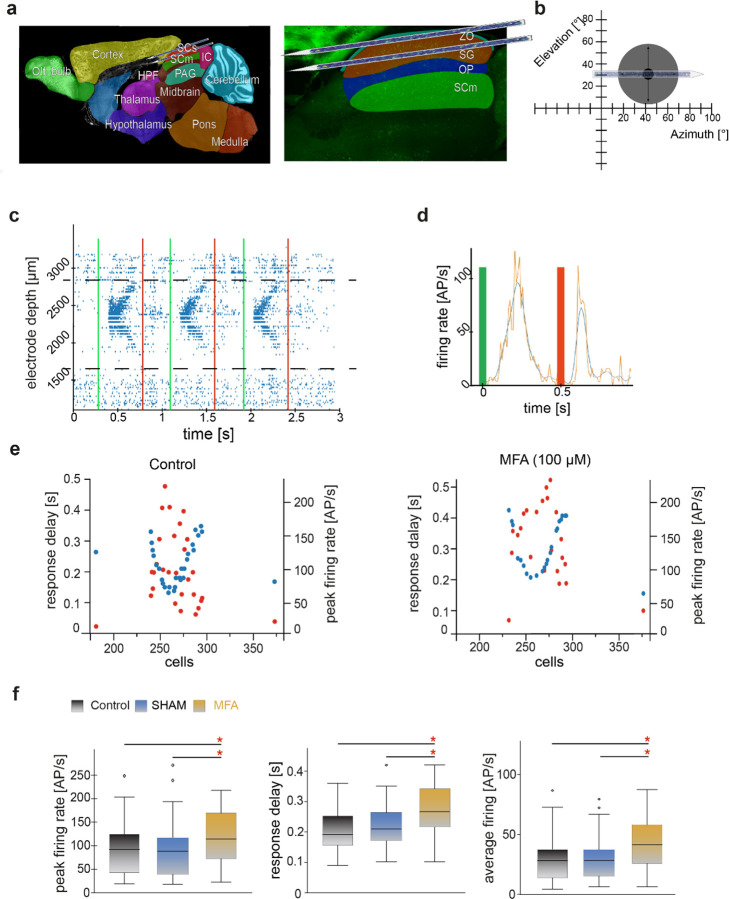
In vivo Electrophysiological SC recordings. **a)** Parasagittal brain sections showing representative probe insertion sites from two experiments. Fluorescent labeling: Thy1-GCaMP3 (green), parvalbumin (PV, blue), SMI-32 (red). **b)** Schematic illustrating sequential activation of SC neurons along the probe during presentation of an expanding visual stimulus. c) Multiunit recordings showing raster plots of SC neuronal activity during looming stimulation. Vertical lines indicate stimulus onset (green) and offset (red). **d)** Peristimulus time histogram (PSTH) of a representative SC neuron showing increased firing after stimulus onset and offset. **e)** Scatter plots showing response latency (blue) and peak firing rate (red) of SC neurons recorded along the probe during looming stimulation. Left, control (SHAM); right, gap junction blockade (MFA eye drops). Neurons near the center of the retinotopic representation showed the shortest response delays. **f)** Quantification of response parameters under control, SHAM, and gap junction blockade conditions. Gap junction blockade altered response latency (control 0.192 ± 0.074, sham-treated 0.21 ± 0.072, MFA 0.27 ± 0.089) and firing rate (peak firing frequency - control 80.88 ± 63.77, sham-treated 74.77 ± 57.97, MFA 97.2 ± 60.34; mean firing frequency - control 25.87 ± 18.52, sham-treated 25.34 ± 18.16, MFA 34.6 ± 23.45). Shown are mean values (line), 25% and 75% quartiles (boxes), data range (whiskers).

Retinal gap junction blockade changed the pattern of looming-evoked responses in retinorecipient SC neurons. To see if the gap junction enhanced retinal coding of looming stimuli has functional consequences we performed NeuroPixel electrophysiological recordings in awake, head-restrained wild-type mice (N = 9) (Fig. 4a) to determine whether a pharmacological gap junction blockade in the retina affects the activity of SC neurons. To record responses to overhead stimuli, we targeted the retinotopically matched region of the SC; probes were inserted into the left caudal SC (0.5 mm lateral to the midline), oriented parallel to the surface to maximize coverage of ventral retinal projections. This orientation allowed for the examination of a horizontal visual field band spanning 20–30° on the right side of the upper visual field and reliable detection of numerous SC neurons responsive to looming stimuli (35 ± 20 cells per experiment (Fig. 4b).

The light-evoked responses of recorded SC neurons reflected the temporal dynamics of the “looming” stimulus; initially only a small group of SC neurons were activated followed by an increasing number of neurons along the probe were recruited as the spot stimulus expanded (Fig. 4c). Concurrently, previously activated neurons became silent, suggesting that most recorded SC neurons respond primarily to dynamic changes within their receptive fields rather than to static stimuli (Fig. 4d). Like observations from targeted tOFFα RGC recordings, the spatial position of the stimulus correlated with SC neuron response latencies, producing a characteristic ‘U’-shaped response delay curve (Fig. 4e). Interestingly the response amplitude of SC cells also varied with the location resulting in an inverse ‘U’ shaped curve of peak firing frequency curve. This pattern indicated that SC neuron response amplitudes decreased progressively toward the peripheral regions of the visual field under looming stimulus conditions (Fig. 4e).

To investigate if retinal gap junction activity influences the firing patterns of postsynaptic SC neurons, animals (n = 9) were treated with the general gap junction blocker MFA. Head-locked mice were anesthetized using isoflurane and 20 μL of 100 μM MFA eyedrops (dissolved in Systane Ultra) were administered topically to both eyes. In addition to untreated controls and MFA-treated groups, SHAM-treated animals (eye drops without MFA) were utilized as well. Changes in the kinetic parameters of control, SHAM-treated and gap junction blocked SC neuron light responses were compared. Notably, SC responses to the “looming” stimulus persisted even after pharmacological blockade of retinal gap junctions. However, detailed analysis revealed three distinct gap junction inhibition initiated changes in response patterns: *(i)* a subset of SC neurons that were responsive under control conditions became inactive when retinal gap junctions were blocked; *(ii)* a second group of SC neurons exhibited activation only after gap junction inhibition; and *(iii)* approximately 50% of SC neurons were responsive under both conditions but demonstrated significant alterations in their response kinetics. Specifically, this third SC neuron group displayed a significant increase in response latency to the “looming” stimulus following MFA treatment (p < 0.05, Mann–Whitney U test), whereas SHAM-treated animals did not exhibit such changes (Fig. 4e, f). Interestingly, the MFA treatment also led to a significant enhancement in response amplitude, as reflected by both peak firing frequency (p < 0.05, Mann–Whitney U test) and mean firing frequency (p < 0.05, Mann–Whitney U test).

While the precise network mechanisms underlying these changes remain to be elucidated, these results suggest that signaling through retinal gap junctions influence SC neuron response kinetics, the pattern, and magnitude of SC responses recruited during looming stimulation.

#### Retinal Gap Junction Perturbation Selectively Impairs Looming-evoked Escape

**Figure 5:**
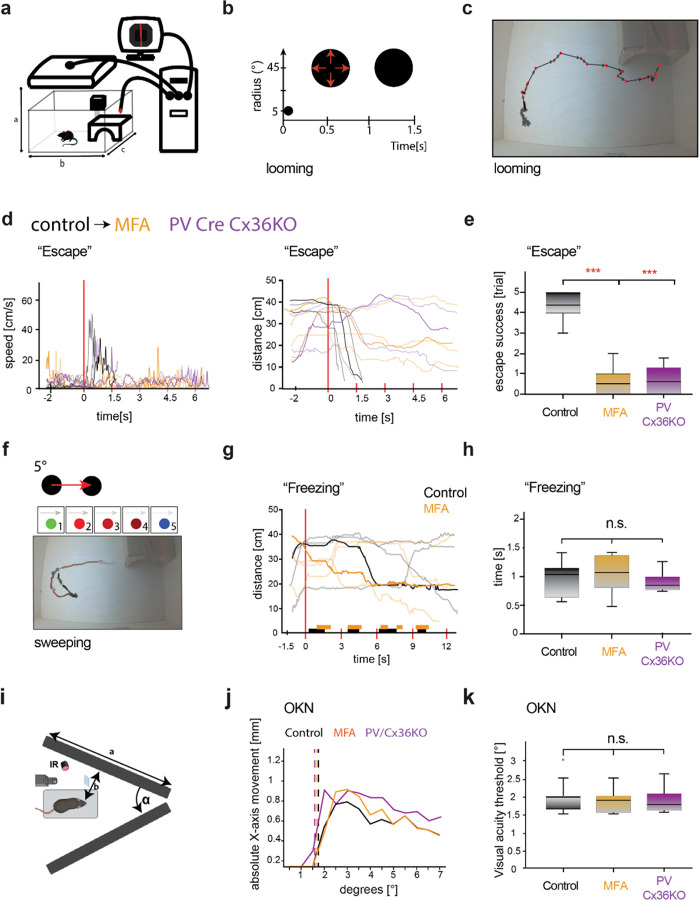
Looming Stimulus Evoked Escape Behavior is Dependent on Retinal Gap Junctions **a)** Schematic of the behavioral setup. An expanding dark spot was presented from an overhead monitor in an arena containing a shelter. Animal trajectories were tracked using DeepLabCut. **b, c)** Time course of the expanding stimulus (b) and representative frame showing mouse trajectory during a trial (c). **d)** Behavioral responses to looming stimulation (n = 8 mice). Left, locomotor speed aligned to stimulus onset (t = 0). Right, distance to shelter. **e)** Number of successful escape responses per session. Gap junction blockade with intraocular MFA reduced escape responses (p < 0.001, Wilcoxon signed-rank test). PV/Cx36KO mice showed reduced escape responses compared to wild-type controls (p < 0.001, Mann–Whitney U test). **f)** Laterally moving stimulus used to evoke freezing behavior. **g)** Freezing responses in control and MFA-treated mice. **h)** Average freezing response time elicited by the sweeping stimulus. No significant differences were observed between control, MFA-treated and PV/Cx36KO animals (Ctrl: 1.05±0.37s, MFA: 1.10±0.37s, PV/Cx36KO 0.9±0.15s; p > 0.05, Wilcoxon signed-rank test, Mann–Whitney U test). **i)** Schematic of the optokinetic reflex (OKR) setup used to assess visual acuity. Head-fixed mice were placed on a running wheel between two monitors displaying moving gratings. Eye movements were recorded using infrared illumination. **j)** Quantification of reflexive pupil tracking under control, gap junction blockade (MFA), and PV/Cx36KO conditions. **k)** Visual acuity thresholds were not significantly different between control, MFA-treated, and PV/Cx36KO mice (Ctrl: 1.8±0.26°, MFA: 1.9±0.30°, PV/Cx36KO: 1.85±0.33°; p > 0.05, Wilcoxon signed-rank test, Mann–Whitney U test). Shown are mean values (line), 25% and 75% quartiles (boxes), data range (whiskers).

We found that a pharmacological retinal gap junction blockade reduced looming-evoked escape behavior. To test whether the gap junction mediated looming code contributes to escape behavior, we conducted a series of behavioral experiments. Mice were placed in a custom-designed arena containing a shelter and an overhead monitor that displayed visual stimuli simulating an looming predator, an expanding dark spot (Fig. 5a), which has been commonly used to evoke innate escape responses (from 0° to 50° in 0.5s)^2–4,6,22^ (Fig. 5a, b). Stimuli were presented five times per session, across five sessions per animal. Each trial was triggered only when mice entered a designated area distal to the shelter (Fig. 5a, c).

Under control conditions, mice consistently responded to the looming stimulus with immediate flight toward the shelter. Escape performance was quantified using a location-time map (n = 8 mice, including only animals showing consistent escape under control) and the speed-time graph. The average reaction time was 0.53 ± 0.17 seconds, and the maximum escape speed reached 44.3 ± 7.58 cm/s, with escape behavior observed in 4.38 ± 0.78 trials per session (Fig. 5d; **Supplementary Video 1.)**. Subsequently, the same animals were treated with the gap junction blocker MFA (100 μM) administered extra-ocularly *via* a Systane Ultra eye drop formulation. After a minimum 30-minute incubation period, mice were retested under identical conditions. Pharmacological gap junction blockade in the eyes markedly reduced escape performance. Most mice failed to initiate flight behavior, showing escape in only 0.57 ± 0.76 trials per session. Furthermore, their movement speed dropped significantly, with a maximum speed of just 5.57 ± 3.2 cm/s post-stimulus (Fig. 5d, e; **Supplementary Video 2**). These deficits were statistically significant for both hiding frequency and escape speed (p < 0.001, Wilcoxon signed-rank test).

Based on the above findings, we sought to attribute the diminishing escape behavior to tOFFα RGC cell gap junction network disturbance. Therefore, we utilized PV/Cx36KO mice (n = 6) and repeated the behavior experiments using the above protocol. PV/Cx36KO mice showed escape response in only 0.7 ± 0.71 trials compared to the 4.4 ± 0.95 successful trials observed in control tests (Fig. 5e; **Supplementary Video 3**). This indicated that, besides pharmacological gap junction blockade, the genetical closure of Cx36 gap junctions in a subset of RGCs, including tOFFα RGCs significantly decreased the looming driven escape behavior (p **<** 0.001, Mann–Whitney U test).

To rule out the effect of the eyedrop vehicle in the observed behavioral deficits a subgroup of animals (n = 5) underwent a standard SHAM treatment (eyedrop without MFA). The results demonstrated no statistically significant differences in escape performance between the control and SHAM-treated groups (p > 0.05, Wilcoxon signed-rank test; **Supplementary Fig. 2g**), respectively. To rule out the effect of salience or habituation on the observed changes we repeated the test in reversed order (n = 6) as well. In this design, mice received MFA treatment *via* eyedrop prior to the initial test, and control (washout) measurements were conducted only after a 3-hour recovery period (sufficient time to reverse the pharmacological effect in 5 out of 6 mice). The results were consistent with those of the initial tests, mice showed a significant MFA induced reduction in the observed escape behavior (p < 0.05, Wilcoxon signed-rank test; **Supplementary Fig. 2h**).

To determine whether the MFA treatment reflected a general visual impairment, we conducted additional control experiments. In the first test, we evaluated freezing behavior evoked by a laterally moving dark stimulus, a response that engages retinal circuits distinct from those involved in looming detection^1,22^. These experiments were conducted in the same arena used for looming testing (Fig. 5a, f). Mice were exposed to repeated lateral motion stimuli while their distance from the shelter was continuously recorded (Fig. 5f). Freezing episodes, characterized by the cessation of exploratory movement, were identified by plateaus in the distance-time curves, indicating minimal locomotion (Fig. 5g, h). We observed that control, MFA-treated (n = 8) and the selective Cx36 KO (n = 6) animals reliably exhibited freezing behavior in response to the lateral motion stimulus. Subsequent analyses indicate no significant difference in freezing incidence or duration between groups (Ctrl vs MFA: p > 0.05 Wilcoxon signed-rank test; Ctrl vs PV/Cx36KO: p > 0.05 Mann–Whitney U test). These findings show that responses to lateral motion stimuli were not affected by MFA treatment.

To assess visual acuity and depth perception a modified visual cliff paradigm was employed. Both the platform and the pit floor were covered with a checkerboard pattern, with differing check sizes to enhance depth perception for animals with normal vision (**Supplementary Fig. 2a**). The two regions (each 0.16 m^2^) were covered with transparent glass, allowing free exploration while preserving the visual illusion of depth. We quantified exploration time and walking distance across each region. Both control and MFA-treated mice exhibited normal exploratory-behavior, preferring peripheral zones along the walls and showed selective avoidance of the pit region and entering the central area only over the platform (**Supplementary Fig. 2a, b**). The time distribution confirmed a strong preference for the platform (~ 80:20 ratio). Importantly, this behavior was unchanged by MFA treatment (n = 6), with no significant difference observed between control and treated animals (**Supplementary Fig. 2c**; p > 0.05, Wilcoxon signed-rank test). These results indicate that visual acuity and depth perception required for the visual cliff test are preserved following retinal gap junction blockade *via* MFA.

Finally, to test if the visual acuity of mice was altered in the various test conditions, we recorded optokinetic reflex responses (Fig. 5i; **Supplementary Fig. 2d, e)** to sequentially increasing grating sizes (0.2° – 7°). Mice were head fixed onto a voluntary running wheel and the stimuli were presented on two monitors facing the mice (Fig. 5i, the two monitors were slanted at 11° degrees relative to each other), while the pupil movements were recorded and analyzed using DeeplabCut (**Supplementary Fig. 2d, e)**. The visual acuity was determined as the visual degree threshold where the pupil tracking showed a positive optokinetic response (Fig. 5j; **Supplementary Fig. 2f)**. We found that neither the pharmacological gap junction blockade using MFA (n = 5) nor the selective Cx36 knockout (n = 6; PV/Cx36KO) alters visual acuity significantly (Fig. 5k; Ctrl vs MFA: p > 0.05 Wilcoxon signed-rank test; Ctrl vs PV/Cx36KO: p > 0.05 Mann–Whitney U test).

Collectively, the above results indicate that the retinal gap junction blockade selectively impairs loomingevoked escape, in contrast all other tested visually guided functions were retained.

## DISCUSSION

In this study, we found that detection of looming objects is not limited to individual tOFFα RGCs but emerges from the coordinated activity of a broader population of RGCs. Gap junction-mediated coupling amplifies this population response by priming neighboring tOFFα RGCs and extending looming-related activity beyond the receptive field of a single neuron. Disrupting this coupling reduces population recruitment, alters activity patterns in retinorecipient neurons of the SC, and weakens escape behavior while sparing other visual functions.

### RGC Gap Junctions Serve Neighbor Priming

Wang et al. observed that targeted ablation of tOFFα cells ceased the escape reflex thus it is a key RGC subtype for the detection of looming objects^3^. This subtype’s essential role is due to its looming discrimination ability. However, the receptive field of tOFFα cells has an average diameter of 200 μm, covering an area of ~ 10° within the visual field, so a tOFFα cell isolated from its neighbors is not suitable for detecting looming object exceeding this size.

We presented evidence here that the light response of tOFFα cells reflects the kinetics of the looming object whose motion was initiated in the middle of their receptive field. Moreover, tOFFα cells that lacked direct stimulation, due to their distal location relative to the stimulus, also produced a light response. Interestingly, this effect was specific to looming stimulation, but stationary or lateral moving stimuli failed to initiate it. This indicates the presence of a very effective filter mechanism that, only upon “looming” stimulation of a single tOFFα RGC, passes the activation towards neighboring RGCs. We believe that, as reported previously, this filter is served by the rapid inhibition from AII amacrine cells that relies on gap junction connections^4^. However, this mechanism only ensures looming selectivity but does not contribute to the intercellular lateral spreading of information found in our experiments.

### Study Constraints and Context

Based on our presented results, the origin of the excitatory priming input remains uncertain, as it may arise from electrically coupled RGCs, ACs, or a combination of both. The observation that gap junction blockade not only eliminated neighbor priming but also reduced the central tOFFα RGC activity suggests that the priming signal originates from a gap junction coupled source with a substantially overlapping receptive field. Because neighboring RGC receptive fields are physically offset, the most likely source is electrically coupled ACs that possess wide-field dendritic arbors. Moreover, their radial dendritic structure^17,19,25–29^ makes them ideal for detecting expanding “looming” motion and processing related signals. By sharing the excitatory input (type 3a bipolar cells) with tOFFα RGCs^28,30^, these ACs likely amplify the looming signal and thus can contribute to tOFFα RGC responses of both direct receptive field stimulation as well as neighbor priming. At this point we believe that gap junction coupled ACs play a crucial role in the observed neighbor priming effect, however, this hypothesis needs further testing.

Another limitation of this work is that Cx36 gap junctions are not selectively eliminated in tOFFα RGCs of the PV/Cx36KO mouse line as about 20% of mouse RGC population (8 subtypes)^31^ express parvalbumin and thus affected in this GMO line. For this reason, the PV/Cx36KO results support our pharmacological findings and narrow the pool of potentially involved RGCs to a subset, but they do not provide subtype specificity. Therefore, future studies utilizing tOFFα RGC specific Cx36KO mice should reinforce our present findings.

Finally, the MFA and quinine pharmacological results in the in vitro MEA recordings, the in vivo SC recordings and the behavioral tests are not subtype specific. However, our targeted tOFFα RGCs patch-clamp recordings in a combination with Ca^++^ -imaging allowed for the selective gap junction blockade of tOFFα RGCs while electrical synapses remained intact in the circuitry. Therefore, these latter experiments yield RGC subtype specific results thereby providing one explanation to the underlying circuit mechanism, while the rest of the experiments show the large-scale effect of retinal gap junction loss incorporating the loss of neighbor priming. Future studies using a tOFFα RGC specific GMO would enable a more precise investigation and help address these limitations.

### Retinal Gap junction Communication Affects the Activity of Retinorecipient SC Circuits and Enhances Visually Guided Behavior

MEA recordings show that looming is encoded by a heterogeneous RGC population: while rare tOFFα RGCs (~ 1%) uniquely discriminate looming from lateral motion, most responsive cells are non-tOFFα types, reflecting mixed looming-sensitive and -selective signals. Consistent with this, SC recordings reveal widespread activation, indicating distributed population coding across retina and SC, with tOFFα pathways forming a specialized subset. Overall, these findings identify a population-level mechanism in which gap junction–mediated coupling coordinates multiple RGC subtypes, extending the spatial range of looming detection while preserving selectivity and linking retinal dynamics to SC activity and behavior.

The tOFFα RGC network is specialized to discriminate looming objects from static images or lateral moving objects^4^, which is essential for animals to avoid looming predators or sources of danger. The ability to tell looming and sweeping predator motion apart is vital for prey animals, as the two stimuli initiate fundamentally different defense mechanisms^1^. This places tOFFα RGCs and their local retinal network at the center of efforts to understand motion processing within visual circuits. Our study here demonstrated that besides stimulus discrimination the tOFFα RGC microcircuit participates in the process with another circuit element, electrical synapses, that spatially extend looming-related signaling thereby strengthening escape behavior. Therefore, based on the presented results we conclude that electrical synapses convert looming detection into a network-level computation and recruits neighboring tOFFα RGCs to amplify threat signals and drive escape behavior

## MATERIALS AND METHODS

### Animals and preparation

Adult (P20<) C57BL6 (n= 39), PV/Cx36KO (n= 15) and Thy1-GCaMP3 (n=15; JAX strain #017893) mice were used in this study. The PV/Cx36KO mouse line was used to selectively disrupt connexin-36 (Cx36)– mediated gap junction coupling in a subset of parvalbumin (PV)–expressing RGCs, including tOFFα RGCs^4^. This conditional knockout line was generated by crossing Cx36^flx/flx mice (gift from Petri Ala-Laurila’s lab), in which exon 2 of the Gjd2 (Cx36) gene is flanked by loxP sites^32–34^, with PV-Cre mice (Jackson Laboratory strain #017320). Offspring were genotyped for the presence of the Cre recombinase transgene and the floxed Cx36 allele according to standard Jackson Laboratory protocols. Animals homozygous for the floxed Cx36 allele and positive for PV-Cre expression (Cx36^flx/flx; PV- Cre^+^33^) were used as PV-specific Cx36 knockout (PV/Cx36KO) mice in all experiments. After overnight dark adaptation, animals were put under deep anesthesia using Forane (4%, 0.2ml/l) and terminated through cervical dislocation. Dissection and experimentation were carried out in mammalian Ringer’s solution 125 mM NaCl, 3 mM KCl, 2 mM CaCl_2_, 1 mM MgCl2, 25 mM NaHCO3, 1.25 mM NaH2PO4, 10 mM glucose, pH 7.4)^35^ under dim red illumination. The eyes and the retina were removed and hemisected anterior to the ora-serrata. In the single-cell extracellular recordings were carried out with tungsten microelectrodes, anterior optics and the vitreous humor were removed, and the resultant retina-eyecup was placed in a superfusion chamber. In MEA or patch clamp (PC) experiments, the retina was completely isolated from the eyecup and placed directly atop the array or a filter paper (Millipore) to further transfer under the PC electrophysiology setup. All animals were treated in accordance with the ARVO Statement for the Use of Animals in Ophthalmic and Vision Research. Maintenance and animal housing were all carried out in accordance with the local Animal Welfare Committee guidelines and regulations (University of Pécs: BA/73/00504-5/2021, BA02/2000-27/2024; KU Leuven: 165/2018). All efforts were made to minimize pain and discomfort.

### Skull-thinning and head-posting

The animals were anesthetized with ketamine, systemic (buprine) and local (naropine) anesthetics were used. The fur and skin were removed from the top of the skull, subsequently the exposed skin and muscles were covered with Vetbond (3M) glue. A metal head-post was attached to the disinfected and cleaned skull with dental cement (Superbond C&B). The skull was then thinned in a rectangular shape on the left cerebral hemisphere (1mm laterally to the left of the midline, 0.5mm to the right lateral of the midline, −2.87mm – −4.67mm from the bregma), the thinned skull was temporarily covered using Smooth-ON Standard-set body double quick-setting silicone. Only after three days of recovery were further experiments performed. In the recovery period, the weight of the animals was checked daily, and a systemic analgesic was applied 12, 24, 36 hours after the intervention.

### Extracellular electrophysiology

Low-resistance voltage clamp (Loose Patch) - Patch Clamp (PC) measurements were performed, detailed in the next chapter. As a second extracellular recording approach, 60- channel Multielectrode (MEA) systems (Multi Channel Systems MCS GmbH, Reutlingen, Germany) were used to record the firing activity of retinal ganglion cells, and the recordings were recorded with the McRack software (Multi Channel Systems MCS GmbH, Reutlingen, Germany). In the third group of experiments, a BioCAM-X highdensity multielectrode (HD-MEA; 4096 channels) system and BrainWave software were used for data collection (3Brain AG, Zurich, Switzerland). In the pharmacological experiments, picrotoxin (PTX; 50 μM), meclofenamic acid (MFA; 40 μM), quinine (100 μM) and L-2-amino-4-phosphonobutyric acid (L-AP4, 50 μM) were used separately or in combination.

### Patch clamp measurements and intracellular delivery of dye molecules

Voltage-clamp -Patch Clamp (PC) recordings were performed using an Axopatch 200B PC amplifier (Axon Instruments Inc., Union City, CA, USA) and ECS-filled PC pipettes (≈20 MΩ; borosilicate glass, 1.5/0.84 mm ID/OD, WPI) in low resistance voltage clamp mode configuration (loose Patch Clamp). Signals were digitized using a Digidata 1440A ADC (Axon Instruments, Inc.) and recorded using WinWCP software (John Dempster, University of Strathclyde, Glasgow, UK). Electrodes used for tracer electroporation were filled with intracellular solution (ICS; 20–30 MΩ borosilicate glass pipette, 1.5/0.84 mm ID/OD, WPI). Electrodes were pulled with a P-87 micropipette puller (Sutter Instruments, Novato, CA, USA). ICS contained 125 mM potassium gluconate, 8 mM NaCl, 0.6 mM MgCl2, 1 mM EGTA, 10 mM HEPES, 2 mM Mg-ATP, and 0.4 mM Na-GTP- t at pH 7.3 (adjusted with KOH) and supplemented with a combination of 0.5% A568-hydrazide and either 4% Neurobiotin (NB) or 0.1% serotonin^26,36^ to inject target cells by electroporation [NB: +65 mV pulses 1 Hz on (V = −50 mV; R = 90 MΩ); serotonin: −65 mV pulses at 1 Hz (V = 15 mV; R = 90 MΩ)]. In the PC recordings, the pharmacological agents were added to the Ringer solution and filled into the Patch pipette.

### In-vivo electrophysiology

Three days after skull thinning, the animals were fixed with the head-post (Thorlabs plate clamp) on a 15 cm diameter Styrofoam ball maintained with compressed air in front of a screen. Neuropixel 1 probes (Imec, Belgium), coated with fluorescent dye (DIi, Thermofisher), were placed in the brain at 25° relative to the skull, at the border of the inferior colliculus and SC, 0.5 mm laterally from the midline. After reaching the appropriate depth, we rested for 20–30 minutes before starting the recording. Artificial cerebrospinal fluid (150 mM NaCl, 5 mM K, 10 mM D-glucose, 2 mM NaH2PO4, 2.5 mM CaCl2, 1 mM MgCl2, 10 mM HEPES with NaOH adjusted to pH 7.4) was to cover the exposed brain area and protect it from drying.

The analog signal was recorded at 30 kHz using the Neuropixel head amplifier (Imec), base station (Imec) and Kintex-7 KC705 FPGA (Xilinx). SpikeGLX software was used to select recording electrodes, set gain corrections, monitor online recordings, and save data. The timing of the visual stimulation was recorded via the digital port of the base station.

### Light stimulus sequences

#### Patch Clamp – PC:

Light stimulus sequences for patch clamp and MEA recordings were programmed in the free cross-platform software PsychoPy (Peirce et al., 2019), and then a high-resolution LED projector delivered them directly to the surface of the retina through an ND2 filter. With the help of PsychoPy software, we generated the stimulus sequence, and for the timing, we sent a signal to an Arduino uno via the serial command line, so that it generates a TTL signal to serve as an analog input for the MEA PCI card or the PC A/D converter.

**Figure 7:**
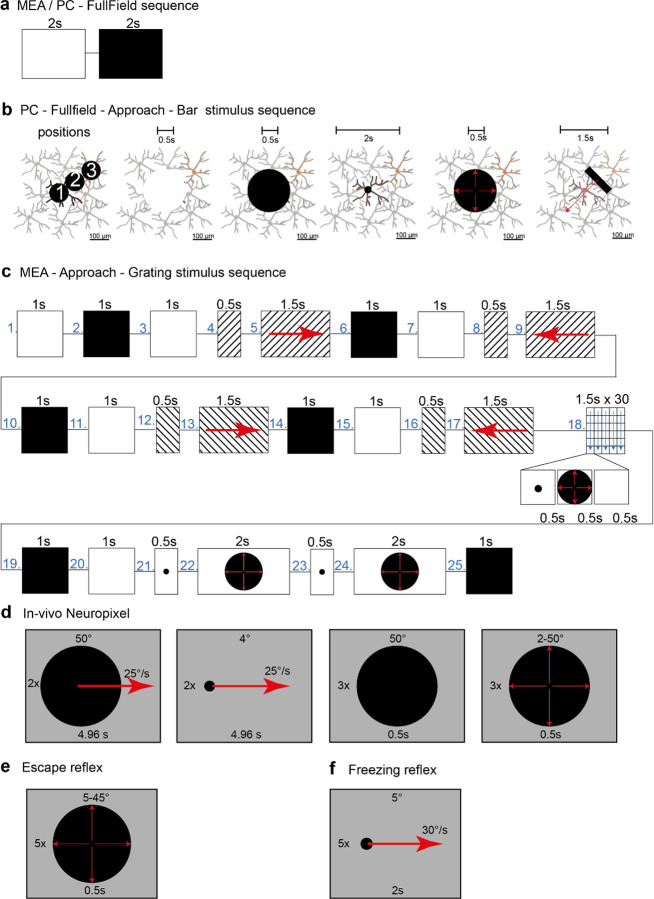
Light stimulation sequences. The figure shows light stimulation sequences applied to different experimental paradigms. **a)** To determine non-specific light response polarity and kinetics, 2s black (OFF) and 2s white (ON) stimuli filling the entire visual field were used in the MEA and PC experiments. **b)** Schematic diagrams show the stimulus sequence used in PC experiments in 3 positions (1. fixed tOFFα cell body (red), 2. halfway between fixed and adjacent tOFFα cell (orange), 3. adjacent tOFFα cell body); red arrows show the movement of the direction, the lines above the pictograms represent the passage of time; scale: 100 μm. **c)** The panel shows the sequence used for the multielectrode experiments (blue numbers: stimulus number); the red arrows show the direction of movement, the banding is equidistal 100 μm black and white bars represent a static or moving stimulus; the time of the stimuli is visible above the pictograms; at step 18, increasing stimuli were shown to the retina as a 5×6 square grid sequence, the order of the sequence is shown by blue arrows. **d, e, f)** Pictograms show the stimuli used for the head-mounted in-vivo Neuropixel and the freely moving in-vivo behavioral tests, in all cases a gray (Gv: 125) background was used; the red arrows show the speed of the movement, the dimensions proportionally represent the dimensions expressed in arc minutes (top of the figures), the number of repetitions immediately after each other is shown on the left side of the images, the speed of the lateral movement is shown on the right side, the length of the individual stimuli is shown at the bottom of the figures.

- To check the identity of the tOFFα RGCs, we used a black and white and looming object stimulus fillingthe entire visual field. During full visual field stimulation, white (2s, gray value - gv: 256) and black illumination (2s, gv: 0) were alternating. For looming stimuli, a 40 μm black circle was projected over the soma for 2 s. The diameter then increased from 40 μm to 240 μm in 0.5 s (400 μm/s) (Fig. 7a).
- The following stimulus sequence was used for kinetic and priming mechanism experiments-established by changing the epicenter position of the looming object: i) 240 μm white spot (0.5s), ii) 240 μm white spot (0.5s), iii) 40 μm black spot (2s), iv) black spot expanding from 40 to 240 μm (400 μm/s, 0.5s), v) moving black band 40 μm thick and 240 μm wide (1.5s). This projector sequence was projected into three positions: 1) The primary cell body, 2) between the neighboring cell and the primary cell, 3) focusing on the neighboring cell body (Fig. 7b).

#### Multielectrode array- MEA:

 We focused the image of the projector on the surface of the MEA with a Tamron 70–300mm F/4–5.6 Di LD Macro camera lens, whose sharpness was adjusted using a Hayear 51MP HDMI USB camera (pixel size: 1.335μm × 1.335μm). The stimulus sequence included 1) a black full-field stimulus, 2) a white full-field stimulus, 3) a stationary bar stimulus (in 4 directions), 4) a moving bar stimulus (in 4 directions), 5) an increasing stimulus appearing sequentially in a 5×6 square grid (300μm × 300μm) (40μm – 0.5s, stimulus increasing from 40μm to 240μm – 0.5s), 6) increasing spot in the center of the visual field (40μm – 0.5s, stimulus increasing from 40μm to 800μm – 2s) (Fig. 7c).

#### In-vivo head-fixed Neuropixel electrophysiology:

The stimulus sequence was programmed with the StimPy Python add-on and projected at a distance of 30 cm from the head-mounted mouse. Four stimulus types were used in random order: 1) 50° progressive black spot (4.96s, 25°/s), 2) 4° progressive black spot (4.96s, 25°/s), 3) 50° black spot (0.5s), 4) Black spot expanding from 2° to 50° (0.5s). The stimuli were indicated by the alternation of white (ON) and black (OFF) spots appearing in the corner of the screen, which was detected with a photodiode through the A/D converter. (Fig. 7d)

#### Behavioral tests:

To examine the reflexes to a looming object (5°−45°, 0.5s) and a moving object (5°, 30°/s, 1s), a 38cm × 30cm monitor was placed horizontally above an arena at a height of 30cm and the two stimuli were shown on it. (Fig. 7e, f)

### Behavioral tests

#### Visual cliff:

The visual cliff experiment was performed in an arena built specifically for this purpose, the schematic top view of which is shown in **Supplementary Fig. 2**. The wooden arena has a 15 cm high rim and a 50×63 cm transparent glass bottom. The space is divided into two halves by the pattern under the glass. On the right side, the closer (glass surface) checkerboard pattern (“ledge”) appears larger, while the checkerboard pattern further (30 cm) on the left side (“cliff”) appears smaller. The size of the squares in both areas was the same (2.54 × 2.54 cm). Adequate lighting of the arena was set to avoid reflections on the glass as much as possible. The mice were naively placed in the middle of the disinfected/deodorized glass surface without habituation. The experiments were recorded with a GoPro Hero 6 camera. The position of the mouse was detected with the DeepLabCut pattern recognition software (at 8 points: nose, right ear, left ear, neck, middle of the back, base of the tail, middle of the tail, end of the tail), and then the time spent on both sides was analyzed in Python. For gap junction inhibition, 100 μM MFA was dissolved in Systane eye drops. After isoflurane anesthesia, 20 μl per drop was placed on the eyes and we waited for the animal to wake up and blink to help the active ingredient diffuse to the eye^37^. The mice were tested after half an hour of incubation.

#### Visual acuity assessment based on the optokinetic reflex:

Visual acuity was assessed using the optokinetic reflex (OKR), an involuntary eye movement elicited by moving visual patterns^38^. Mice were head-fixed on a voluntary running wheel, while visual stimuli were presented on two monitors positioned in front of the animal (Fig. 5i). The monitors were oriented with a mutual angle of 11° to provide a wide visual field. Pupil movements were recorded using a Hayear 51 MP HDMI USB camera (pixel size: 1.335 μm × 1.335 μm) and analyzed using DeepLabCut (Supplementary Fig. 2d, e). The pupil contour was defined by eight landmark points (nasal, dorsonasal, dorsal, dorsotemporal, temporal, ventrotemporal, ventral, and ventronasal), which were manually annotated and used to train the DeepLabCut network. For each video frame, the pupil center position was calculated using a custom Python script, and horizontal (x-axis) pupil displacement was extracted during stimulus presentation. Absolute x-axis movements were quantified to capture the magnitude of optokinetic tracking. Visual stimuli consisted of horizontally drifting black-and-white square-wave gratings with sequentially increasing bar widths ranging from 0.2° to 7°, moving at a constant speed of 3°/s across the visual field. Visual acuity was defined as the smallest grating bar width (visual angle) that elicited a reliable optokinetic tracking response, determined from the inflection point (“elbow”) in the pupil displacement curve (Fig. 5j; Supplementary Fig. 2f).

#### Escape and freezing reflexes:

Escape and freezing reflexes were induced as described in the light stimulation section. The mice were placed in a disinfected/deodorized box with dimensions of 30cm × 46cm × 20cm, equipped with a hiding place, 2 hours daily for 2 days for habituation. Three treatment types were used: 1) control - ∅ eye drops, 2) MFA - 100 μM MFA + 20 μl eye drops, 3) sham-treated - 20 μl eye drops. After the appropriate treatment, the animal was placed in the arena, when most of the time they immediately went to the hiding place. The stimulus started when the animals were in the other half of the arena as seen from the hiding place. The experiments were recorded with a GoPro Hero 6 camera. The position of the mouse at all times was detected with the DeepLabCut pattern recognition software (at 8 points: nose, right ear, left ear, neck, middle of the back, base of the tail, middle of the tail, end of the tail), and then the mouse's movement speed and the momentary distance from the hiding place were analyzed in Python.

### Data analysis

For the 64-channel Multielectrode extracellular leads, action potentials were sorted using Spike2 (CED, Cambridge, UK) and Offline Sorter (Plexon Instruments, Dallas, TX, USA), while the Hearding-Spikes sorter was used for HD-MEA recordings^39^, the results of the Neuropixel recordings were analyzed with the Kilosort sorting software. Peristimulus time histograms (PSTH) measuring transients were generated in NeuroExplorer (Plexon Instruments, Dallas, TX, USA) or using a Python script using the Scikit-learn package^40^. Gaussian smoothing (filter size: 3) applied to each. Transience values were calculated using the PSTHτ method^41^, where PSTHτ measures the time required for the firing frequency to decrease to 1/e of the peak firing amplitude. SPSS (v19, IBM, Armonk, NY, USA). OriginPro 18 (Origin Lab Corp., Northampton, MA, USA) was used for statistical analysis. Data are presented as mean ± SD for descriptive purposes unless stated otherwise. Statistical comparisons were performed using nonparametric tests (Wilcoxon signed-rank test for paired data and Mann–Whitney U test for independent groups), which do not assume normality.

### Post-hoc immunohistochemistry

To visualize cells loaded with NB and serotonin, samples were incubated in oxygenated Ringer's solution for at least 15 min after injections. The tissues were then fixed in 4% paraformaldehyde solution for 15–25 minutes, washed with phosphate buffered saline (PBS) and CTA (5% Chemiblocker, 0.5% Triton X-100, 0.05% sodium azide in PBS- in) were blocked overnight and then incubated overnight in CTA-diluted Streptavidin Cy5 (Thermo Fisher Scientific; 1500-fold dilution). Samples containing serotonin-filled cells were processed for serotonin immunohistochemistry using anti-serotonin antiserum (Sigma-Aldrich, St. Louis, MO, USA, catalog number: S5545, 2000-fold dilution) for 2 days, and after thorough washing (4x) with PBS- in), DyLight^™^ 405 AffiniPure Goat Anti-Rabbit (111-475-003, 500x) secondary antibody was added to the tissue for fluorescence visualization. Washed retinal samples were placed on slides mounted in VectaShield (Vector Laboratories, Newark, CA, USA) and coverslipped for microscopic examination. In addition to tracer visualization, we also performed other immunohistochemical experiments, which are summarized in Table 1.

### Confocal microscopy and image analysis

Retinal samples were scanned using a Zeiss LSM710 confocal microscope with a 20x (Z = 1 μm; Zeiss W Plan-Apochromat 20/1.0) and 63x objective (Z = 0.5 μm; Zeiss Plan Apochromat 63/1.4) high-resolution and normalized laser. Minor adjustments to image brightness and contrast were made in FIJI - ImageJ, NIH, and Adobe Photoshop CC (Adobe Systems Inc., San Jose, CA, USA).

### Reconstruction of injected, coupled arrays

Confocal optical sections were imported into FIJI scientific image processing software. After using the Simple Neurite Tracer^42^ plug-in, we were able to trace all the extensions of individual cells. First, the cell body was marked in concentric circles from the first to the last optical section in which it was present. Then, starting from the soma, we followed the primary extensions to the very first branching point, then from the branching point to the end of the visible extensions, thus marking the entire dendritic tree. After selecting all the extensions belonging to each cell, we performed the filling function and exported the result as a grayscale image. The grayscale images were used for further analysis, and 3D models were generated for visualization using the FIJI three-dimensional (3D) display plugin.

### Supplementary Files

This is a list of supplementary files associated with this preprint. Click to download.

- SupplementaryFigurelegend.docx
- LoomingescapeMFA2x.mp4
- LoomingescapeControl2x.MP4.mp4
- LoomingescapePVCx36KO2x.MP4.mp4

## Figures and Tables

**Figure 6:**
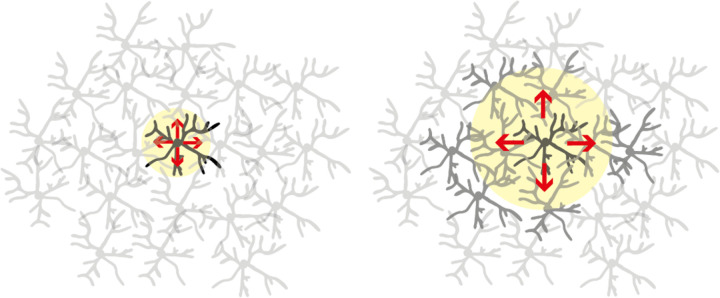
Visual Function of Gap Junction Mediated Priming in the Coupled tOFFα RGC system **a)** Schematic showing a lattice of tOFFα RGCs (grey neuron outlines). In the case of gap junction closure (left panel) the presented looming visual stimulus (indicated by the outward pointing red arrows) only the central cell is activated, thus further size growth over the single neuron receptive field does not evoke additional activation due to inadequate stimulus component (laterally moving edge) and/or fast inhibition of neighboring cells. On the other hand, when gap junctions formed by tOFFα RGCs serve the priming of neighboring cells (right panel) the system overcomes this inhibiting effect and helps recruit a population of tOFFα RGCs to detect the looming stimulus beyond the single cell receptive field.

**Table 1 |:** Antibody list. List of primary and secondary antibody concentrations utilized.

Antibodies	Host	Supplier	Cat. nr. / RRID	Concentration
**anti-HCN4**	Rabbit	Alomone	APC-052/AB_2039906	2000x
**anti-CaBP5**	Rabbit	SySy	475 002/AB_2924962	2000x
**anti-Chat**	Rabbit	Thermofisher	PA5-29653/AB_2547128	1500x
**DyLight^™^ 405 anti-Rabbit**	-	Jackson	711-475-152/AB_2340616	500x
**DyLight^™^ 405 anti-Guineapig**	-	Jackson	706-475-148/AB_2340470	500x
**DyLight^™^ 405 anti-Mouse**	-	Abcam	ab175658/AB_2340813	500x
**anti-MouseCy3**	Donkey	Jackson	715-165-150/AB_2340813	500x
**anti-Rabbit 488**	Horse	Jackson	308-545-003/N/A	500x
**Streptavidin Cy5**	-	Sigma	PA45001/N/A	1500x

## HIGHLIGHTS

- Looming stimuli recruit a distributed population of retinal ganglion cells
- Gap junctions enable the priming of neighboring transient OFF-alpha cells
- Retinal gap-junction perturbation reshapes looming responses in the colliculus
- Electrical synapses support looming-evoked escape behavior
